# Diagnostic potential of near-infrared Raman spectroscopy in the stomach: differentiating dysplasia from normal tissue

**DOI:** 10.1038/sj.bjc.6604176

**Published:** 2008-01-15

**Authors:** S K Teh, W Zheng, K Y Ho, M Teh, K G Yeoh, Z Huang

**Affiliations:** 1 Bioimaging Laboratory, Division of Bioengineering, Faculty of Engineering, National University of Singapore, Singapore 117576, Singapore; 2 Department of Medicine, Yoo Loo Lin School of Medicine, National University of Singapore and National University Hospital, Singapore 119260, Singapore; 3 Department of Pathology, Yoo Loo Lin School of Medicine, National University of Singapore and National University Hospital, Singapore 119074, Singapore

**Keywords:** dysplasia, near-infrared Raman spectroscopy, optical diagnosis, stomach, principal components analysis, linear discriminant analysis

## Abstract

Raman spectroscopy is a molecular vibrational spectroscopic technique that is capable of optically probing the biomolecular changes associated with diseased transformation. The purpose of this study was to explore near-infrared (NIR) Raman spectroscopy for identifying dysplasia from normal gastric mucosa tissue. A rapid-acquisition dispersive-type NIR Raman system was utilised for tissue Raman spectroscopic measurements at 785 nm laser excitation. A total of 76 gastric tissue samples obtained from 44 patients who underwent endoscopy investigation or gastrectomy operation were used in this study. The histopathological examinations showed that 55 tissue specimens were normal and 21 were dysplasia. Both the empirical approach and multivariate statistical techniques, including principal components analysis (PCA), and linear discriminant analysis (LDA), together with the leave-one-sample-out cross-validation method, were employed to develop effective diagnostic algorithms for classification of Raman spectra between normal and dysplastic gastric tissues. High-quality Raman spectra in the range of 800–1800 cm^−1^ can be acquired from gastric tissue within 5 s. There are specific spectral differences in Raman spectra between normal and dysplasia tissue, particularly in the spectral ranges of 1200–1500 cm^−1^ and 1600–1800 cm^−1^, which contained signals related to amide III and amide I of proteins, CH_3_CH_2_ twisting of proteins/nucleic acids, and the C=C stretching mode of phospholipids, respectively. The empirical diagnostic algorithm based on the ratio of the Raman peak intensity at 875 cm^−1^ to the peak intensity at 1450 cm^−1^ gave the diagnostic sensitivity of 85.7% and specificity of 80.0%, whereas the diagnostic algorithms based on PCA-LDA yielded the diagnostic sensitivity of 95.2% and specificity 90.9% for separating dysplasia from normal gastric tissue. Receiver operating characteristic (ROC) curves further confirmed that the most effective diagnostic algorithm can be derived from the PCA-LDA technique. Therefore, NIR Raman spectroscopy in conjunction with multivariate statistical technique has potential for rapid diagnosis of dysplasia in the stomach based on the optical evaluation of spectral features of biomolecules.

Gastric cancer is currently the fourth most common malignancy, and also the second leading cause of cancer deaths in humans worldwide (Axon, 2006; Clark *et al*, 2006). In Singapore, despite a falling incidence rate, gastric cancer still remains the fourth most common cancer (Teh *et al*, 2002). Many of these patients will die mainly because of nodal and metastatic disease present at the time of initial diagnosis. Early detection and localisation with immediate removal and treatment of premalignant lesions (e.g., dysplasia) (Clark *et al*, 2006) is crucial to improving patients' survival. However, early identification of dysplasia in the stomach can be very difficult to detect by conventional diagnostic methods such as white-light endoscope, as the white-light endoscopy heavily relies on the visual observation of gross morphological changes of pathologic tissues, leading to a poor diagnostic accuracy.

In the past decade, optical spectroscopic methods such as Raman spectroscopy, which makes use of inelastic light scattering process to capture ‘fingerprints’ of specific molecular structures and conformations of a given tissue or disease state, have been comprehensively investigated for cancer and precancer diagnosis and evaluation in humans (Frank *et al*, 1995; Mahadevan-Jansen and Richards-Kortum, 1996; Gniadecka *et al*, 1997; Mahadevan-Jansen *et al*, 1998a; Bakker Schut *et al*, 2000; Shim *et al*, 2000; Stone *et al*, 2000; Caspers *et al*, 2003; Huang *et al*, 2003). Near-infrared (NIR) Raman spectroscopy has certain advantages over Fourier transform IR spectroscopy in tissue diagnosis, such as relative insensitivity to water, and a deeper penetration in the tissue using NIR excitation light. As such, NIR Raman spectroscopy has received great interest for *in vitro* and *in vivo* diagnosis of malignancies in a variety of organs (Mizuno *et al*, 1994; Frank *et al*, 1995; Gniadecka *et al*, 1997; Mahadevan-Jansen *et al*, 1998a; Shim *et al*, 2000; Stone *et al*, 2000; Caspers *et al*, 2003; Huang *et al*, 2003). These investigations show that specific spectral features of Raman spectra could be used to correlate with the molecular and structural changes of tissue associated with neoplastic transformation (Mahadevan-Jansen and Richards-Kortum, 1996; Gniadecka *et al*, 1997; Mahadevan-Jansen *et al*, 1998a; Stone *et al*, 2000; Huang *et al*, 2003). For instance, using NIR Raman technique, the diagnostic sensitivity and specificity of 82 and 92%, respectively, can be achieved for differentiation between precancerous and benign cervical tissues *in vitro* (Mahadevan-Jansen *et al*, 1998a). Near-infrared Raman spectroscopy has also been applied for *in vivo* precancer and cancer diagnosis and detection of organs such as cervix, skin, colon, and oesophagus (Mahadevan-Jansen *et al*, 1998b; Shim *et al*, 2000; Huang *et al*, 2001; Utzinger *et al*, 2001). The Raman spectroscopic characterisation and discrimination of malignancy in the stomach have also been investigated (Ling *et al*, 2002; Stone *et al*, 2002; Kumar *et al*, 2007). However, to date, application of Raman spectroscopy on early diagnosis of gastric precancer (dysplasia) has not yet been reported in detail in literature.

Despite the great advantages that NIR Raman spectroscopy could offer, there are technical challenges to overcome. For instance, achieving a high signal-to-noise (S/N) ratio, while avoiding interference from silica Raman signals in a rapid manner can be difficult for *in vivo* tissue Raman measurements (Bakker Schut *et al*, 2000; Huang *et al*, 2001; Utzinger *et al*, 2001). This is because tissue Raman scattering is inherently very weak, and the fibre-optic probes used to collect *in vivo* signals exhibit strong silica Raman scattering in the fingerprint region. Also, the integration times and irradiance powers for *in vivo* Raman measurements must be limited for practical and safety reasons. Furthermore, Raman spectral differences are usually subtle with apparent spectral overlappings and variations in intensity between different tissue types, and thus developing effective diagnosis algorithms are highly required for effective tissue classification (Bakker Schut *et al*, 2000; Shim *et al*, 2000; Huang *et al*, 2003, 2004; Molckovsky *et al*, 2003; Lau *et al*, 2005). The primary aims of this study were to characterise Raman properties of gastric tissues and to assess the feasibility of using a rapid fibre-optic NIR Raman spectroscopy for precancer diagnosis of gastric tissue. Both the empirical approach and the multivariate statistical techniques, including principal components analysis (PCA) and linear discriminant analysis (LDA), were employed to develop effective diagnostic algorithms for differentiations between normal and dysplasia tissue in the stomach.

## MATERIALS AND METHODS

### Raman instrumentation

The instrument used for tissue Raman spectroscopic studies has been described in detail elsewhere (Huang *et al*, 2001). Briefly, this system consists of a 785-nm diode laser, a transmissive imaging spectrograph with a Kaiser holographic grating, an NIR-optimised back-illuminated, deep-depletion charge-coupled device (CCD) detector (Princeton Instruments, Trenton, NJ, USA), and an in-house developed fibre optic Raman probe. The 785-nm laser is coupled to a 100-*μ*m core diameter fibre (NA=0.22) and the fibre is connected to the Raman probe via an SMA connector. The Raman probe was designed to maximise the collection of tissue Raman signals while reducing the interference of Rayleigh scattered light, fibre fluorescence, and silica Raman signals. One optical arm of the probe consists of a collimating lens, a bandpass filter (785±2.5 nm, Chroma Technology Corp., Rockingham, VT, USA), and a focusing lens to deliver the laser light onto the tissue. The other arm of the probe equipped with collimating and refocusing lenses and a holographic notch plus filter (optical density >6.0 at 785 nm; Kaiser) is used for collecting tissue Raman signals. The holographic notch filter was placed between the two lenses to block the Rayleigh scattered excitation laser light while passing the frequency-shifted tissue Raman signal. The refocusing lens then focused the filtered beam onto the circular end of the fibre bundle (58 × 100 *μ*m core diameter fibres, NA=0.22). Tissue Raman photons collected by the fibre bundle in the Raman probe are fed into the entrance of the transmissive spectrograph along a parabolic curve, and the holographic grating disperses the incoming light onto the liquid nitrogen-cooled CCD array detector controlled by a principal component (PC) (Huang *et al*, 2001, 2003). The tissue Raman spectra associated with autofluorescence background are displayed on the computer screen in real time and can be saved for further analysis. The system acquired Raman spectra over the wavenumber range of 800–1800 cm^−1^, and each spectrum was acquired within 5 s with light irradiance of 1.56 W cm^−2^. The spectral resolution of the system is 4 cm^−1^. All wavelength-calibrated spectra were also corrected for the wavelength dependence of the system using a standard lamp (RS-10; EG&G Gamma Scientific, San Diego, CA, USA).

### Gastric tissue samples

**Figure 1:**
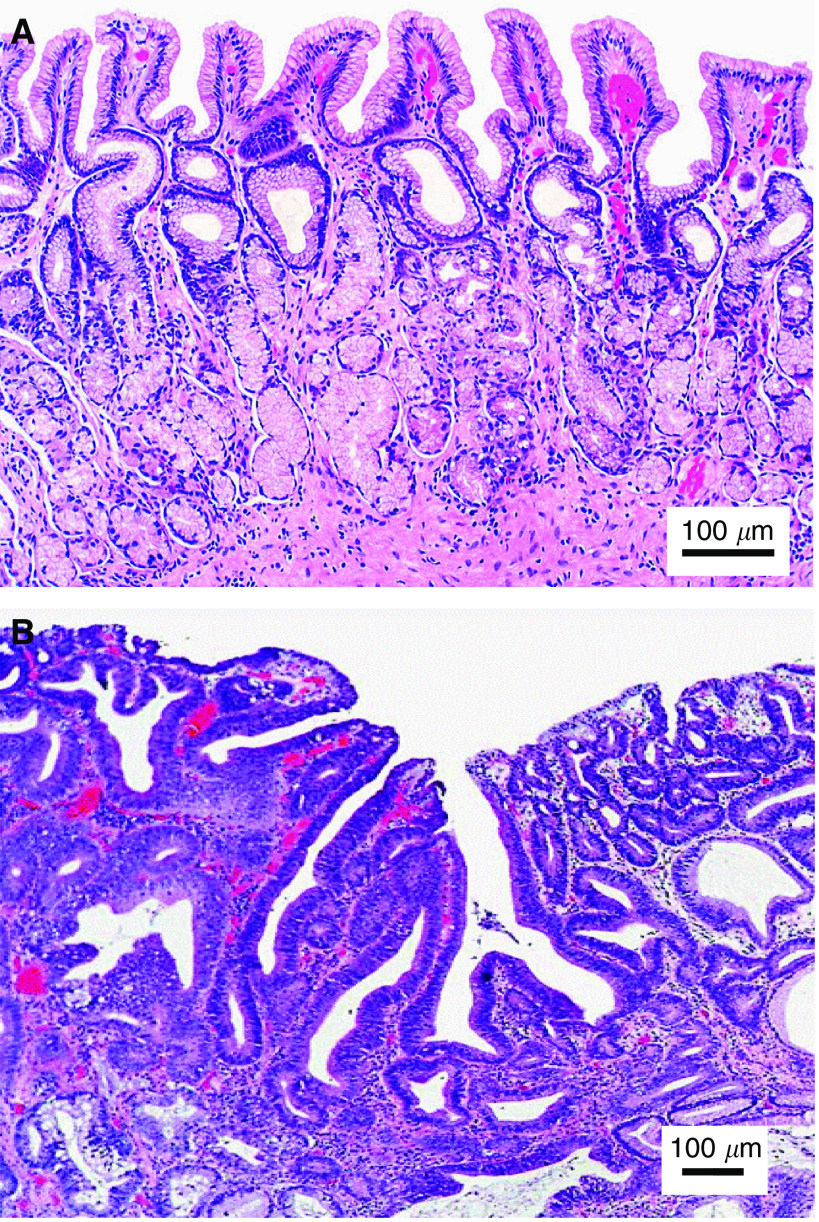
Photomicrographs of the haematoxylin and eosin (H&E)-stained tissue sections of gastric tissues (**A**) normal and (**B**) dysplasia (high-grade dysplasia of the antrum). Scale bar: 100 *μ*m.

A total of 76 gastric tissue samples were collected from 44 patients (21 men and 23 women with a median age of 62 years) who underwent gastrectomy or endoscopic biopsies with clinically suspicious lesions. All patients preoperatively signed an informed consent, permitting the investigative use of the tissues, and this study was approved by the Ethics Committee of the National Healthcare Group (NHG) of Singapore. After biopsies or surgical resections, tissue samples were immediately sent to the laboratory for Raman measurements. After spectral measurements, the tissue samples were fixed in 10% formalin solution and then submitted back to the hospital for histopathologic examination. The histopathogical examinations were conducted by a specialist gastrointestinal pathologist, and the results showed that among the 76 homogenous gastric tissue samples with clearly defined pathologies, 55 tissue specimens were normal, and 21 were dysplasia (8 low-grade and 13 high-grade dysplasia). Figure 1 shows the comparison of haematoxylin and eosin (H&E)-stained tissue sections of normal and dysplastic gastric tissues, illustrating the crowding of irregularly shaped glands with branching and prominent nuclear abnormalities (including irregular and thickened nuclear membranes and irregular chromatin) in dysplasia mucosa. Note that the gastric tissue samples were approximately 3 × 3 × 2 mm in size, and the 785-nm laser light with a beam size of 1 mm was focused on the tissue surface to mimic the *in vivo* clinical measurements. The tissue surface measured was then marked and stained for tissue pathology. After comparing with pathologic results, only those Raman spectra that were correctly acquired from the surfaces of gastric tissues were used for data analysis. To reduce the spectral measurement errors in this study, the average spectrum of five repeated Raman measurements on the same tissue site of each tissue sample was used for tissue classification.

We have measured the thickness of various layers of typical normal and dysplastic tissue sections and constructed a gastric tissue model, which consisted of mucosa, submucosa, muscularis propria, and serosa layers with a total thickness of 1.5 mm for normal tissue, while the dysplasia tissue consisted of thickening mucosa, submucosa, muscle propria, and serosa layers with a total thickness of 2 mm (Sabet *et al*, 2003). Optical parameters (absorption coefficient, scattering coefficient, scattering anisotropy, and refractive index) from literature (Bashkatov *et al*, 2007) were composed for these layers to set up a tissue optics model for Monte Carlo simulation of light penetration into these model tissues. The simulation results (data not shown) showed that the 785-nm light penetrated down to about 750 *μ*m beneath the surface of normal tissue, which was within the muscularis propria layer. For dysplasia tissue, the 785-nm light penetrated down to about 780 *μ*m beneath the surface, which was also well within the muscularis propria. It is expected that most of the spectral signals from normal tissue came from mucosa, submucosa layers with some small contributions from the muscularis propria, and serosa layer, because tissue layers closing to the surface encountered stronger excitation light and the generated Raman scattered light was also easier to escape out of the tissue. Similarly, most of the spectral signals from dysplasia came from mucosa, submucosal layers. The exact percentile contributions of various tissue layers can be obtained by more detailed modelling of not only the excitation light propagation but also the Raman scattered light propagation in tissue.

### Data preprocessing

The raw spectra acquired from gastric tissue in the 800–1800 cm^−1^ range represented a combination of prominent tissue autofluorescence, weak tissue Raman scattering signals, and noise. Thus, the raw spectra were preprocessed by adjacent five-point smoothing to reduce noise. A fifth-order polynomial (Huang *et al*, 2003) was found to be optimal for fitting the broad autofluorescence background in the noise-smoothed spectrum, and this polynomial was then subtracted from the raw spectrum to yield the tissue Raman spectrum alone. Each of background-subtracted Raman spectrum was also normalised to the integrated area under the curve from 800 to 1800 cm^−1^ to enable a better comparison of the spectral shapes and relative peak intensities among the different tissue samples.

### Empirical approach

Nonparametric diagnostic algorithms based on peak intensities, spectral bandwidths, and/or peak ratios have been widely employed in literature to correlate the variations of tissue spectra with tissue pathology in a simple and straightforward fashion (Mahadevan-Jansen and Richards-Kortum, 1996; Mahadevan-Jansen *et al*, 1998a; Huang *et al*, 2003). In this study, the empirical diagnostic algorithm based on the ratio of the Raman peak intensity at 875 cm^−1^ for hydroxyproline to the peak intensity at 1450 cm^−1^ for CH_2_ proteins/lipids (Stone *et al*, 2000; Huang *et al*, 2003) was selected for tissue classification. The unpaired Student's *t*-test was used to test the difference of Raman intensity ratio (I_875_/I_1450_) between normal and dysplasia tissues. For the assessment of diagnostic sensitivity and specificity, histopathological results were regarded as the gold standard.

### Multivariate analysis

The high dimension of Raman spectral space (each Raman spectrum ranging from 800 to 1800 cm^−1^ with a set of 544 intensities) will result in computational complexity and inefficiency in optimisation and implementation of the LDA algorithms. As such, PCA was first performed on tissue Raman data set to reduce the dimension of Raman spectral space while retaining the most diagnostically significant information for tissue classification. To eliminate the influence of inter- and/or intra-subject spectral variability on PCA, the entire spectra were standardised so that the mean of the spectra was zero, and the standard deviation (s.d.) of all the spectral intensities was one. Mean centring ensures that the (PCs form an orthogonal basis (Lachenbruch and Mickey, 1968; Devore, 1992). The standardised Raman data sets were assembled into data matrices with wavenumber columns and individual case rows. Thus, PCA was performed on the standardised spectral data matrices to generate PCs comprising a reduced number of orthogonal variables that accounted for most of the total variance in original spectra. Each loading vector is related to the original spectrum by a variable called the PC score, which represents the weight of that particular component against the basis spectrum. Principal component scores reflect the differences between different classes. Unpaired Student's *t*-test (Devore, 1992) was used to identify the most diagnostically significant PCs (*P*<0.05). These significant PC scores are lastly selected as input for the development of LDA algorithms for binary-class classification. Linear discriminant analysis determines the discriminant function that maximises the variances in the data between groups while minimising the variances between members of the same group. The performance of the diagnostic algorithms rendered by the LDA models for correctly predicting the tissue groups (i.e., normal *vs* dysplasia) was estimated in an unbiased manner using the leave-one-sample-out, cross-validation method (Lachenbruch and Mickey, 1968; Dillion and Goldstein, 1984) on all model spectra. In this method, one sample (i.e., one spectrum) was held out from the data set, and the entire algorithm including PCA and LDA was redeveloped using the remaining tissue spectra. The algorithm was then used to classify the withheld spectrum. This process was repeated until all withheld spectra were classified.

To compare the performance of the empirical and multivariate approaches for tissue classification using the same Raman data set, receiver operating characteristic (ROC) curves were generated by successively changing the thresholds to determine correct and incorrect classifications for all tissue samples.

## RESULTS

**Figure 2:**
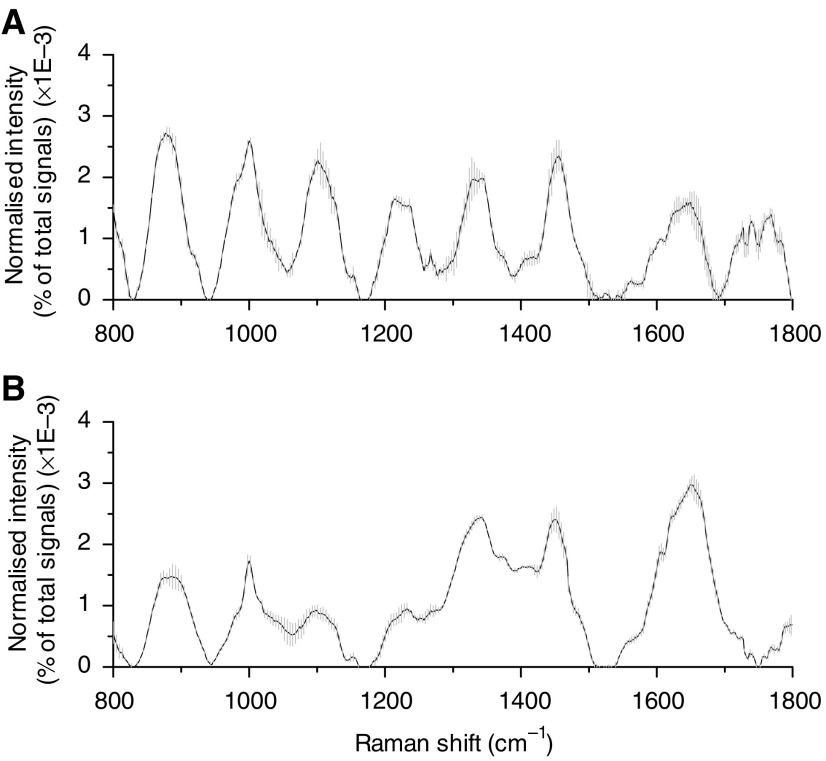
Mean normalised gastric Raman spectra (solid line) ±1 s.d. (grey area) obtained from a normal tissue (**A**) and a dysplasia tissue (**B**) by multiple measurements (*n*=5) at various locations for each sample. Each spectrum was normalised to the integrated area under the curve to correct for variations in absolute spectral intensity. All spectra were acquired in 5 s with 785-nm excitation and corrected for spectral response of the system.

To assess intrasample variability, multiple Raman measurements (*n*=5) on each of normal and dysplasia gastric tissues were made at different locations of the same samples. Figure 2 shows an example of the mean normalised Raman spectra ±1 s.d. measured from a normal (A) and a dysplasia (B) gastric tissue, respectively. The overall spectral intensities varied by 30% about the mean for normal tissue, and by 20% for dysplasia tissue. However, the relative Raman peak heights, shapes, and positions showed little intrasample variability for either normal or dysplasia tissue, indicating the relative homogeneity of tissue samples used in this study.

**Figure 3:**
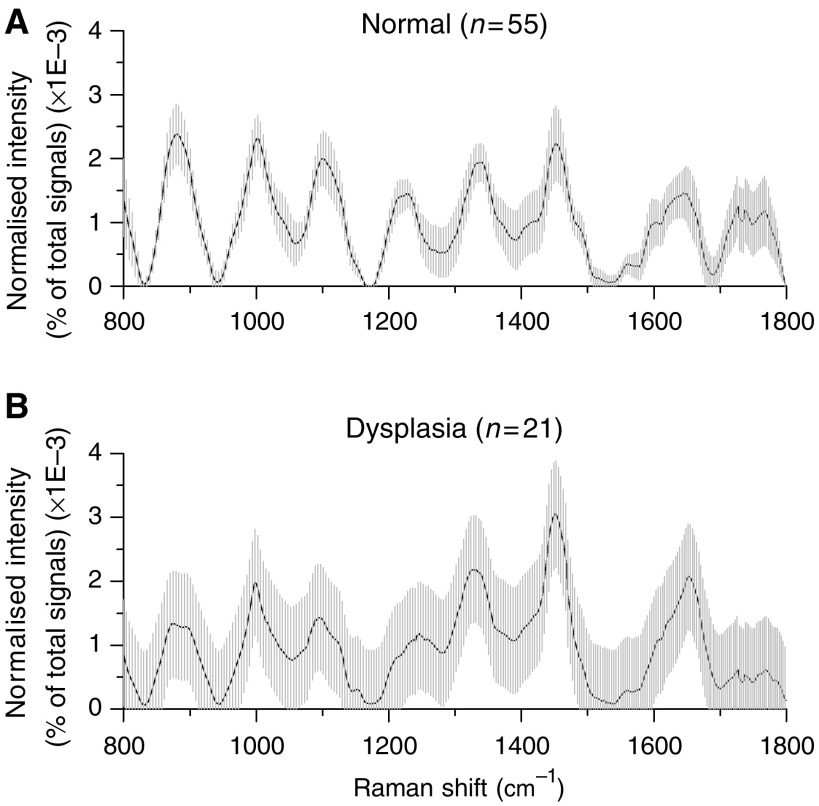
Mean normalised gastric Raman spectra ±1 s.d. (shaded area) from (**A**) normal tissues (*n*=55) and (**B**) dysplasia tissues (*n*=21), illustrating the intensity variations in major Raman peaks of 20–30% for normal tissues whereas of 30–60% for dysplasia tissues.

The intersample variations of all Raman spectra from normal or dysplasia tissues obtained from 44 patients were also studied. Figure 3 shows the mean normalised Raman spectra ±1 s.d. from normal (*n*=55) and dysplasia (*n*=21) gastric tissues. The overall spectral intensities varied by 20–30% about the mean for normal tissue, and by 30–60% for dysplasia tissue. Although there are only some small changes in spectral shapes and Raman peak positions between normal and dysplasia tissue, there is a large overlap in intensity throughout the entire spectral range of Raman spectra between the two tissue types. This indicates a relatively large variability of tissue constituents among different subjects. Hence, there is a need to develop effective diagnostic algorithms for accurate classification of Raman spectra between normal and dysplastic tissues.

**Figure 4:**
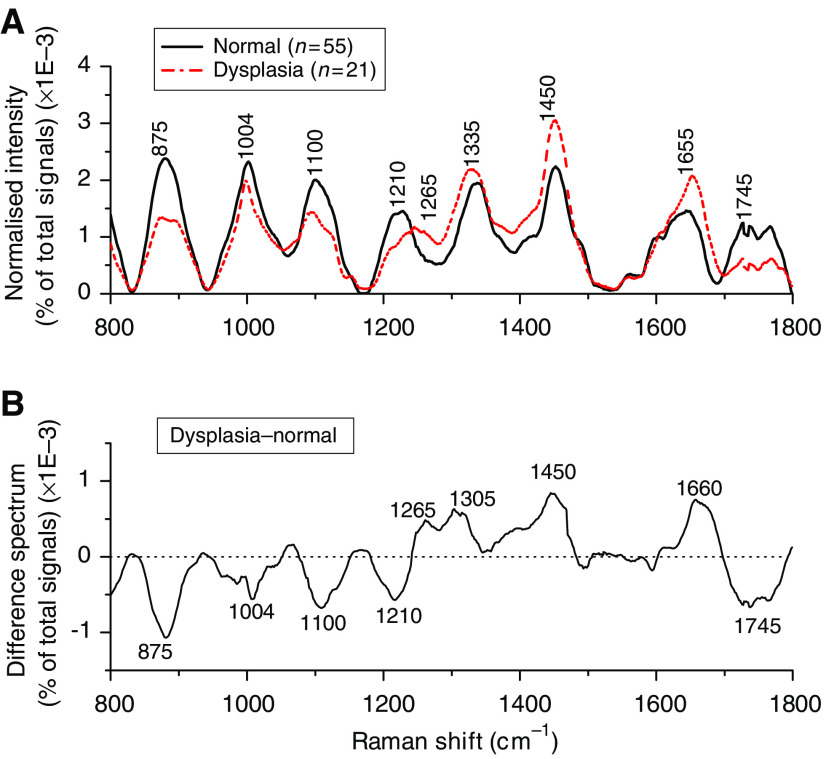
(**A**) Comparison of the mean normalised Raman spectra of normal (*n*=55) and dysplasia (*n*=21) tissues. (**B**) Difference spectrum calculated from the mean Raman spectra of normal and dysplasia tissue (i.e., the mean normalised Raman spectrum of dysplasia tissue minus the mean normalised Raman spectrum of normal tissue).

Figure 4A shows the comparison of mean normalised Raman spectra between normal and dysplasia gastric tissue. The prominent Raman peaks located at around 875, 1004, 1100, 1210, 1265, 1335, 1450, 1655, and 1745 cm^−1^ are observed in gastric tissue, which can be attributed to the biochemical bonds of hydroxyproline, the phenylalanine (an essential amino acid) ring breathing mode, phospholipids (stretching C-C skeletal vibrations in the gauche conformation), tryptophan (an essential amino acid) and phenylalanine stretching (C-C_6_H_5_) mode, amide III (C-N stretching mode of proteins, indicating mainly *α*-helix conformation), CH_3_CH_2_ twisting mode of proteins and nucleic acids, CH_2_ bending mode of proteins and lipids, the protein amide I band (C=O stretching mode of proteins, indicating mainly *α*-helix conformation), and the C=O stretching mode of phospholipids, respectively (Dollish *et al*, 1974; Mizuno *et al*, 1994; Frank *et al*, 1995; Mahadevan-Jansen and Richards-Kortum, 1996; Gniadecka *et al*, 1997; Mahadevan-Jansen *et al*, 1998a, 1998b; Bakker Schut *et al*, 2000; Shim *et al*, 2000; Stone *et al*, 2000, 2002; Huang *et al*, 2001, 2003; Utzinger *et al*, 2001; Caspers *et al*, 2003). The intensity differences between the two tissue types are remarkable (Figure 4B). For instance, dysplasia tissues show lower intensities at 875, 1004, 1100, 1210, and 1745 cm^−1^, while higher at 1265, 1305, 1450, and 1660 cm^−1^, compared with normal tissue. This indicates that there is an increase or decrease in the percentage of a certain type of biomolecules relative to the total Raman-active constituents in dysplasia tissue. There are also obvious changes of Raman peak shifts and bandwidths in the spectral ranges of 1200–1500 cm^−1^ and 1600–1800 cm^−1^, which are related to the amide III and amide I of proteins, CH_3_CH_2_ twisting of proteins/nucleic acids, and C=C stretching of phospholipids for dysplasia. These spectral differences between normal and dysplasia tissues can be viewed more clearly using the difference spectrum as shown in Figure 4B. The difference spectrum reveals the changes of prominent Raman peaks occurring in dysplasia gastric tissue, confirming a potential role of Raman spectroscopy for precancer diagnosis in the stomach.

**Figure 5:**
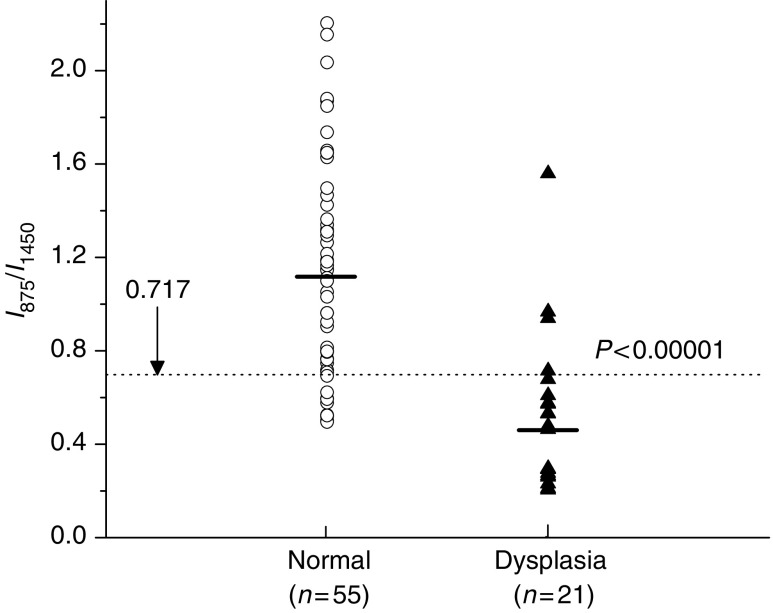
Scatter plot of the intensity ratio of Raman signals at 875 and 1450 cm^−1^, as measured for each sample and classified according to the histological results. The decision line (I_875_/I_1450_=0.717) separates dysplasia tissue from normal tissue with a sensitivity of 85.7% (18/21) and specificity of 80.0% (44/55).

The empirical analysis based on the intensity ratio of prominent Raman bands is employed for tissue diagnosis. Figure 5 shows the scatter plot of the ratio of Raman intensity at 875 cm^−1^ to that at 1450 cm^−1^ grouped according to tissue pathologic types. The mean value (mean±s.d.) of this ratio for normal tissues (1.13±0.46, *n*=55) is significantly different from the mean value for dysplastic tissues (0.52±0.33, *n*=21) (unpaired two-sided Student's *t*-test, *P*<0.00001). The decision line (I_875_/I_1450_=0.717) discriminates dysplasia tissue from normal gastric tissue with a sensitivity of 85.7% and a specificity of 80.0%.

**Figure 6:**
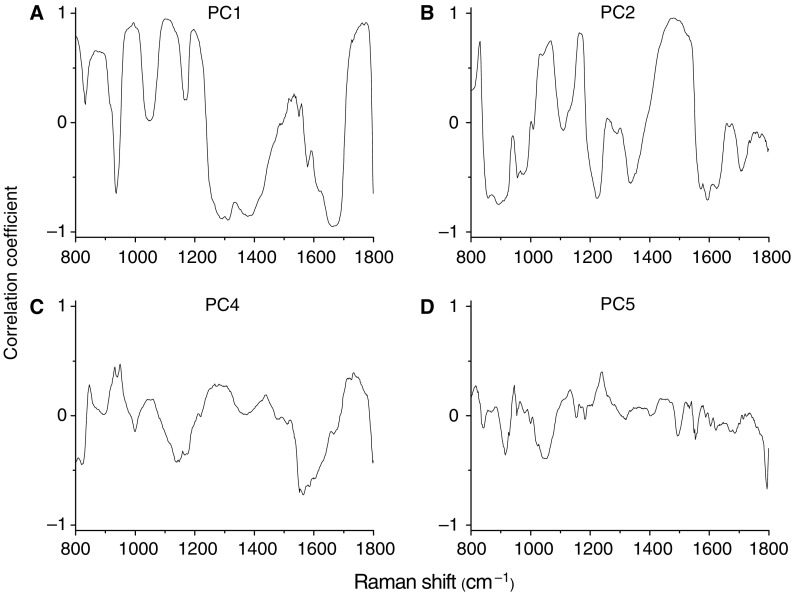
The first four diagnostically significant principal components (PCs) accounting for about 78.5% of the total variance calculated from Raman spectra (PC1 – 42.6%, PC2 – 25.4%, PC4 – 7.9%, and PC5 – 2.6%), revealing the diagnostically significant spectral features for tissue classification.

We also employ the multivariate statistical method (e.g., PCA and LDA) by incorporating the entire Raman spectrum to determine the most diagnostically significant Raman features for improving tissue analysis and classification. Unpaired two-sided Student's *t*-test on the obtained PC scores showed that there were four PCs (PC1, PC2, PC4, and PC5) that were diagnostically significant (*P*<0.05) for discriminating dysplasia tissue from normal tissue. Figure 6 displays the four significant PC scores calculated from PCA on the Raman spectra. The first PC accounts for the largest variance (e.g., 42.6% of the total variance), whereas the successive PCs describe the spectral features that contribute progressively smaller variances. Some PC features (Figures 6A–D), such as peaks, troughs, and spectral shapes are similar to those of tissue Raman spectra in Figure 4.

**Figure 7:**
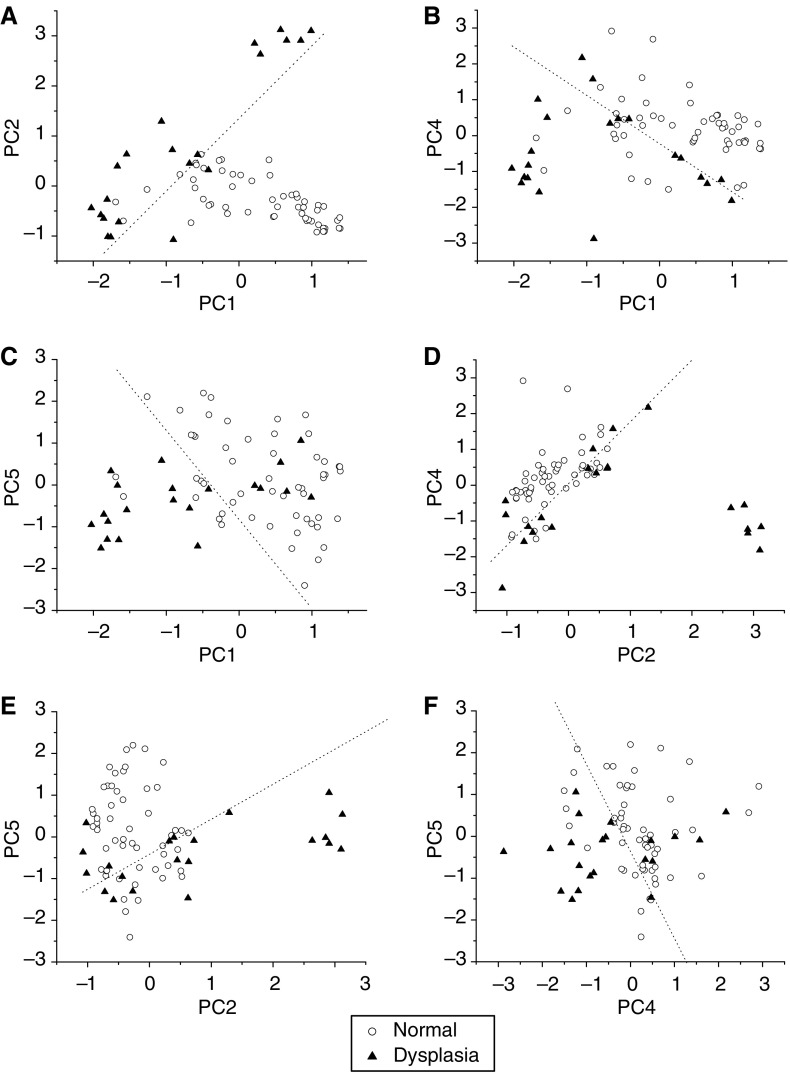
Scatter plots of the diagnostically significantly principal component (PC) scores for normal and dysplastic gastric tissue derived from Raman spectra, (**A**) PC1 *vs* PC2; (**B**) PC1 *vs* PC4; (**C**) PC1 *vs* PC5; (**D**) PC2 *vs* PC4; (**E**) PC2 *vs* PC5; and (**F**) PC4 *vs* PC5. The dotted lines (PC2=1.46 PC1+1.34; PC4=−1.32 PC1+0.94; PC5=−2.16 PC1−0.89; PC4=1.74 PC2+0.12; PC5=0.84 PC2−0.381; and PC5=−2.05 PC4−0.29) as diagnostic algorithms classify dysplasia from normal with sensitivity of 90.5% (19/21), 76.2% (16/21), 71.4% (15/21), 81.0% (17/21), 71.4% (15/21), and 71.4% (15/21); specificity of 90.9% (50/55), 80.0% (44/55), 83.6% (46/55), 80.0% (44/55), 72.7% (40/55), and 72.7% (40/55), respectively. Circle (○): normal; Triangle (▴): dysplasia.

Figure 7 shows the correlations between the diagnostically significant PC scores for normal and dysplastic gastric tissue, illustrating the utility of PC scores for classification of Raman spectra between different tissue types. Normal and dysplasia tissues can be largely clustered into two separate groups based on different combinations of significant PCs, and the corresponding separation lines (i.e., diagnostic algorithms) in Figures 7A–F classify dysplasia from normal tissue with the sensitivity of 90.5%, 76.2%, 71.4%, 81.0%, 71.4%, and 71.4%; specificity of 90.9%, 80.0%, 83.6%, 80.0%, 72.7%, and 72.7%, respectively. These results show that selection of different combinations of significant PCs will give different levels of accuracy for tissue classification.

**Figure 8:**
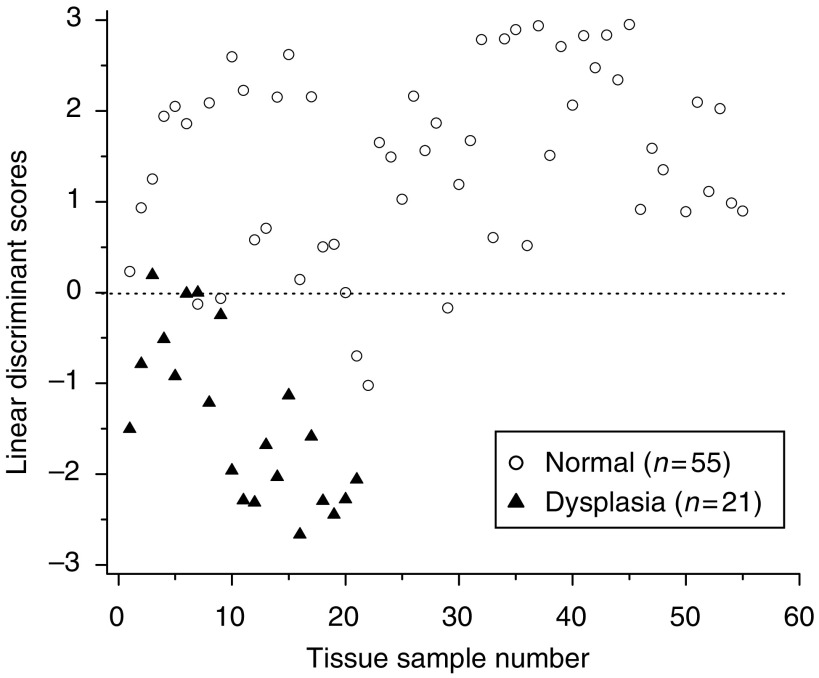
Scatter plot of the linear discriminant scores for the normal and dysplasia categories using the PCA-LDA technique together with leave-one-spectrum-out, cross-validation method. The separate line yields a diagnostic sensitivity of 95.2% (20/21) and specificity of 90.9% (50/55) for differentiation between normal and dysplasia tissue. LDA, linear discriminant analysis; PCA, principal components analysis.

To further improve tissue diagnosis, all the four diagnostically significant PCs were loaded into the LDA model for generating effective diagnostic algorithms for tissue classification. Figure 8 shows the classification results based on PCA-LDA technique together with leave-one-spectrum-out, cross-validation method. The PCA-LDA diagnostic algorithms yielded the diagnostic sensitivity of 95.2% and specificity 90.9% for separating dysplasia from normal gastric tissues.

**Figure 9:**
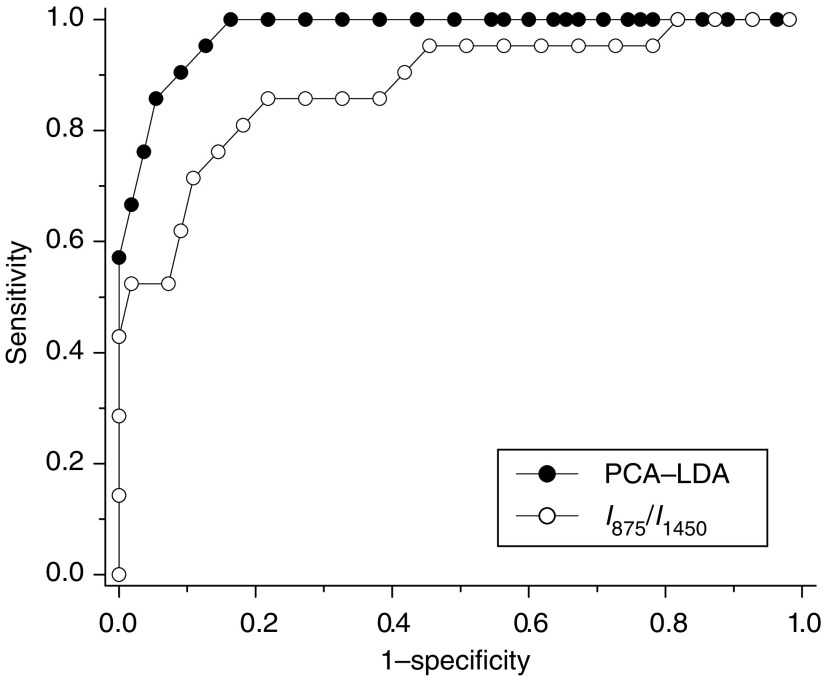
Comparison of receiver operating characteristic (ROC) curves of discrimination results for Raman spectra utilising the PCA-LDA-based spectral classification with leave-one-spectrum-out, cross-validation method and the empirical approach using Raman intensity ratio of I_875_/I_1450_. The integration areas under the ROC curves are 0.98 and 0.88 for PCA-LDA-based diagnostic algorithm and intensity ratio algorithm, respectively, demonstrating the efficacy of PCA-LDA algorithms for tissue classification. LDA, linear discriminant analysis; PCA, principal components analysis.

To evaluate and compare the performance of the PCA-LDA-based diagnostic algorithms derived from all the significant PCs of tissue Raman data set against the empirical approach-based diagnostic algorithm derived from the intensity ratio of I_875_/I_1450_, the ROC curves (Figure 9) were generated from the scatter plots in Figures 5 and 8 at different threshold levels, displaying the discrimination results using both diagnostic algorithms. A comparative evaluation of the ROC curves indicates that PCA-LDA-based diagnostic algorithm gives more effective diagnostic capability for detection of gastric dysplasia from normal gastric tissues, as illustrated by the improvement in the diagnostic sensitivity and specificity. The integration areas under the ROC curves are 0.98 and 0.88, respectively, for PCA-LDA-based diagnostic algorithms and the nonparametric intensity ratio algorithm, respectively. These results demonstrate that PCA-LDA-based diagnostic algorithms that utilised the entire spectral features of Raman spectra yield a better diagnostics accuracy than the empirical approach.

## DISCUSSION

The current gold standard for clinical diagnosis of gastric dysplasia is through histological observation by the pathologist, on the extent of cytological and architectural abnormalities of the histologically prepared tissue samples (Lauwers and Riddell, 1999). These abnormalities involve much molecular alterations, which could also be tapped upon for diagnosis, most importantly during routine endoscopic inspection (Lauwers and Riddell, 1999). Hence, Raman spectroscopy, which is capable of providing rich biochemical and biomolecular information about tissue, may be the promising diagnostic tool to be used for molecular discrimination of gastric dysplasia. However, as gastric dysplasia belongs to part of a widely accepted multistep, continuum progression cascade from normal gastric tissue to adenocarcinoma (Correa, 1988), it implies vague molecular distinction of gastric dysplasia that may render characterisation and discrimination tougher for Raman spectral analysis. As shown in Figure 3, the Raman spectral pattern between normal and dysplastic gastric tissues could be very similar, it is highly desirable to develop robust diagnostic approaches to extract all possible diagnostic information contained in tissue Raman spectra for well correlation with tissue changes associated with neoplastic transformation. Consequently, both empirical and statistical techniques were explored in this study to attain the likelihood of good clinical discriminators of Raman spectra for separation between normal and dysplastic gastric tissues.

The results of this study confirm that there are specific spectral differences in Raman spectra between dysplasia and normal tissue, demonstrating the utility of NIR Raman spectroscopy in gastric precancer detection. For instance, the relative peak intensities at 1450 cm^−1^ (CH_2_ proteins/lipids) and 1305 cm^−1^ (bending mode of CH_3_CH_2_ twisting of protein) (Mahadevan-Jansen and Richards-Kortum, 1996; Stone *et al*, 2000; Huang *et al*, 2003) were found to be higher for dysplasia tissues, indicating the elevated concentration of biomolecules (e.g., histones) due to hyperchromatism in tissue with dysplastic transformation (Lauwers and Riddell, 1999). In addition, there is also a relative increase of amide III band (1265 cm^−1^) and amide I band (1655 cm^−1^) in intensity, suggesting that dysplasia tissue may be associated with an increase in the relative amount of proteins in the *α*-helix conformation. This could be another evidence that there is an increase concentration of histones, the main protein component that makes up the chromatin for dysplasia tissue (Thomas and Prescott, 1977; Huang *et al*, 2005). A shoulder band at 1660 cm^−1^ (amide I, *β*-pleated sheet, and/or random coil conformation) was also revealed in the difference spectra (Figure 4B), suggesting that dysplastic transformation may also be associated with an increase in the relative amount of protein in the *β*-pleated sheet (Huang *et al*, 2003; Stone *et al*, 2004). The appearance of these proteins in the *β*-pleated sheet conformation may signify more chemical interaction between the proteins and the microenvironment occurring in the cells, which could be related to increase of mitotic activity, one of the cellular alteration characteristics of gastric dysplasia (Correa, 1988). On top of all these, the Raman band at 1335 cm^−1^ due to the mixture of biochemicals (nucleic acids and proteins due to extracellular matrix) (Stone *et al*, 2000; Huang *et al*, 2003) showed slightly higher percentage signals for dysplasia tissue, indicating that the percentage of nucleic acid and protein contents relative to the total Raman-active components is also increased in dysplasia tissue. Raman peak intensity at 875 cm^−1^ (hydroxyproline of collagen) was found to be much reduced in dysplastic tissue, and this was probably due to the cytoplasmic mucin depletion and the elevated concentration of metalloproteinase, which cleaved collagen in the stroma layer in gastric dysplasia (Correa, 1988; Georgakoudi *et al*, 2002). On the other hand, the thickening of the epithelium associated with dysplastic progression may attenuate the excitation laser power and also obscure the collagen Raman emission from the deep collagen basal membrane (Badizadegan *et al*, 2004), thus resulting in a much decrease of Raman signals (875 cm^−1^) from dysplasia tissue. In addition, the Raman peaks at 1100 and 1745 cm^−1^ due to phospholipids, and Raman bands for phenylalanine and tryptophan at 1004 and 1210 cm^−1^, respectively, also showed lower percentage signals for dysplasia tissue compared with the normal tissue, suggesting a decrease in the percentage of phospholipids, phenylalanine, and tryptophan relative to the total Raman-active constituents in the dysplasia (Stone *et al*, 2000; Huang *et al*, 2003). The decrease of Raman peak at around 1745 cm^−1^ associated with dysplastic tissue has also been reported in epithelial tissue with malignancies (Ling *et al*, 2002; Huang *et al*, 2003, 2005). Therefore, the distinctive differences in Raman spectra between normal and dysplasia tissue further reinforce that Raman spectroscopy can be used to reveal molecular and cellular changes associated with dysplastic transformation.

To develop simple but effective algorithms for identifying abnormal tissue from normal tissue, the nonparametric empirical approach utilising peak intensity or peak intensity ratio measurements of Raman spectra has been widely applied in a number of organ sites to evaluate variations in the tissue spectra associated with malignant changes (Mahadevan-Jansen and Richards-Kortum, 1996; Utzinger *et al*, 2001; Huang *et al*, 2003). For example, the ratio of intensities at 1655 cm^−1^ (C=O stretching of collagen and elastin) to 1455 cm^−1^ (CH_2_ scissoring of proteins and lipids) has been used to spectrally separate tumours from normal tissues in the brain, breast, colon cervix, and the lung (Utzinger *et al*, 2001; Huang *et al*, 2003), as both bands are sensitive to histological abnormality (Mahadevan-Jansen and Richards-Kortum, 1996). For differentiation of normal and precancerous tissues, other different intensity bands and ratios such as I_1656_, I_1656_/I_1325_, I_1330_/I_1454_ *vs* I_1454_/I_1656_, and I_1336_/I_1250_ had also been reported to be of effective diagnostic algorithms for tissue diagnosis and characterisation (Mahadevan-Jansen and Richards-Kortum, 1996; Huang *et al*, 2003). In this work, selection of different prominent Raman peaks (e.g., peak intensity, bandwidth, and Raman shift) has also been comprehensively explored for gastric tissue classification. On the basis of the difference spectrum between normal and dysplasia tissue (Figure 4B), we found that the nonparametric intensity ratio of Raman peak intensity at 875 cm^−1^ for hydroxyproline to the peak at 1450 cm^−1^ for CH_2_ mode of proteins/lipids was one of the best diagnostic algorithms that yielded a diagnostic sensitivity of 85.7% and a specificity of 80.0% for separating dysplasia from normal tissue. The significant difference of the intensity ratio (I_875_/I_1450_) between normal and dysplasia tissue may reflect the relative changes in the concentration of potential biological markers from cell surface antigens, cytoplasmic proteins and mucin, collagen in the extracellular matrix, enzymes, and hormones in dysplasia (Correa, 1988; Mahadevan-Jansen and Richards-Kortum, 1996). Further investigation also shows that other intensity ratios including the Raman peak intensity band at 1335 cm^−1^ (nucleic acids/proteins) with respect to the Raman peak intensities at 1100 cm^−1^ (phospholipids) and 1745 cm^−1^ (phospholipids) are also statistically significantly different (*P*<0.0001) between normal and dysplasia tissue (data not shown). These ratio values are in agreement with histopathologic studies of grading malignancy by the nucleic acid-to-cytoplasm ratio (Lauwers and Riddell, 1999; Huang *et al*, 2003; Mourant *et al*, 2005). Hence, the above intensity ratios may also potentially be used as diagnostic algorithms for detecting precancer in the stomach.

The simplistic empirical analysis above only employs a limited number of Raman peaks for tissue diagnosis; most of the information contained in the Raman spectra has not been used for spectral analysis. Since biological tissue is complex, it is likely that there are many biochemical species influencing diseases concurrently. Therefore, a multivariate statistical analysis (e.g., PCA and LDA) (Lachenbruch and Mickey, 1968; Deinum *et al*, 1999) that utilises the entire spectrum to determine the most diagnostically significant spectral features may improve the diagnostic efficiency of Raman technique for tissue analysis and classification. As such, PCA-LDA together with cross-validation technique was applied in this work to the NIR Raman spectra acquired for dysplasia tissue identification. The unpaired, two-sided Student's *t*-test identified that only a few PCs (PC1, PC2, PC4, and PC5) contained the most diagnostically significant information (*P*<0.05) for tissue classification. We note that one of the most statistically significant PCs (e.g., PC5) only describes small amount (2.6%) of the total variance. This indicates that some PCs with small variances can still contain the useful diagnostic information for revealing molecular changes with dysplastic transformation. However, since the noise present in weak tissue Raman signals may affect the determination of significant PCs with smaller variances for tissue diagnosis (Sasic, 2001), caution should be taken when acquiring the weak tissue Raman signals. Hence, the rapid fibre-optic Raman system with a high S/N ratio (3.3- to 16-fold improvement) (Huang *et al*, 2001) was employed to obtain high-quality Raman tissue spectra, and an appropriate data preprocessing was also introduced for further reducing the noise interference in PC analysis. The consistency in identifying similar, significant PC scores from run to run during the leave-one-spectrum-out, cross-validation testing suggested that the diagnostic algorithms developed were robust for Raman spectral analysis in this study. To develop effective diagnostic algorithms for tissue classification, all the four diagnostically significant PCs were utilised in the LDA model. The diagnostic sensitivity and specificity of 95.2 and 90.9%, respectively, for identifying dysplasia from normal gastric tissue can be achieved using the PCA-LDA model, which had almost a 10% improvement in diagnostic accuracy compared with the empirical method. Receiver operating characteristic analysis (Figure 8) further confirms that PCA-LDA-based diagnostic algorithms employing the entire spectral features of Raman spectra are more robust and powerful in distinguishing dysplasia from normal tissue.

It should be noted that PCA is primarily for data reduction rather than for identification of biochemical or biomolecular components of tissue. It is usually difficult to interpret the physical meanings of the component spectra. However, with more powerful diagnostic algorithms (e.g., genetic algorithms) (Mountford *et al*, 2001), distinctive spectral regions that are optimal for tissue differentiation may be identified and related to particular biochemical and biomolecular changes (e.g., proteins, lipids, nucleic acids, and carbohydrates) associated with neoplastic transformation. These techniques need a much larger Raman data set for robust diagnostic algorithms development. On the other hand, to further understand the relationships between the dysplasia-related morphologic/biochemical changes and the Raman spectra from tissue that is crucial in establishing confidence in clinicians on the application of rapid fibre-optic Raman spectroscopy for precancer detection, confocal Raman microspectroscopy should be explored on the tissue *in vivo* or *in vitro*, by measuring the complete Raman spectra of specific tissue microstructures, or alternatively by mapping the distribution of some specific Raman peaks or principal components within a tissue, or even mapping the biochemical distribution at different tissue depth for association with tissue histopathology (Caspers *et al*, 2003; Shetty *et al*, 2006). The work in these areas warrants further investigation.

In conclusion, this work shows that significant differences exist in Raman spectra between normal and dysplastic gastric tissue, demonstrating that NIR Raman spectroscopy have the potential to provide diagnostic information necessary for distinguishing precancer from normal tissue. Furthermore, with the development of micron-scale Raman probes for the collection of tissue Raman signals in a few seconds via endoscopy (Shim *et al*, 2000; Hattori *et al*, 2007), NIR Raman spectroscopy could be a potentially clinically useful tool for the rapid and noninvasive early diagnosis of gastric precancer *in vivo* at the molecular level.

## References

[bib1] Axon A (2006) Symptoms and diagnosis of gastric cancer at early curable stage. Best Pract Res Clin Gastroenterol 20(4): 697–708 16997154 10.1016/j.bpg.2006.03.015

[bib2] Badizadegan K, Backman V, Boone CW, Crum CP, Dasari RR, Georgakoudi I, Keefe K, Munger K, Shapshay SM, Sheetse EE, Feld MS (2004) Spectroscopic diagnosis and imaging of invisible pre-cancer. Faraday Discuss 126: 265–279 14992412 10.1039/b305410a

[bib3] Bakker Schut TC, Witjes MJ, Sterenborg HJ, Speelman OC, Roodenburg JL, Marple ET, Bruining HA, Puppels GJ (2000) *In vivo* detection of dysplastic tissue by Raman spectroscopy. Anal Chem 72: 6010–6018 11140770 10.1021/ac000780u

[bib4] Bashkatov AN, Genina EA, Kochubey VI, Gavrilova AA, Kapralov SV, Grishaev VA, Tuchin VV (2007) Optical properties of human stomach mucosa in the spectral range from 400 to 2000 nm: prognosis for gastroenterology. Med Laser Appl 22(2): 95–104

[bib5] Caspers PJ, Lucassen GW, Puppels GJ (2003) Combined *in vivo* confocal Raman spectroscopy and confocal microscopy of human skin. Biophys J 85(1): 572–580 12829511 10.1016/S0006-3495(03)74501-9 PMC1303112

[bib6] Clark CJ, Thirlby RC, Picozzi Jr V, Schembre DB, Cummings FP, Lin E (2006) Current problems in surgery: gastric cancer. Curr Probl Surg 43: 566–570 17000267 10.1067/j.cpsurg.2006.06.003

[bib7] Correa P (1988) A human model of gastric carcinogenesis. Cancer Res 48: 3554–3560 3288329

[bib8] Deinum G, Rodriquez D, Romer TJ, Fitzmaurice M, Kramer JR, Feld MS (1999) Histological classification of Raman spectra of human coronary artery atherosclerosis using principal component analysis. Appl Spectrosc 53: 938–942

[bib9] Devore JL (1992) Probability and Statistics for Engineering and the Science. Pacific Grove: Brooks/Cole

[bib10] Dillion RW, Goldstein M (1984) Multivariate Analysis: Methods and Applications. New York: John Wiley and Sons

[bib11] Dollish FR, Fateley WG, Bentley FF (1974) Characteristics Raman Frequencies of Organic Compounds. New York: Wiley

[bib12] Frank CJ, McCreery RL, Redd DC (1995) Raman spectroscopy of normal and diseased human breast tissues. Anal Chem 67: 777–783 7762814 10.1021/ac00101a001

[bib13] Georgakoudi I, Jacobson BC, Müller MG, Sheets EE, Badizadegan K, Carr-Locke DL, Crum CP, Boone CW, Dasari RR, Van Dam J, Feld MS (2002) NAD(P)H and collagen as an *in vivo* quantitative fluorescence biomarkers of epithelial precancerous changes. Cancer Res 62: 682–687 11830520

[bib14] Gniadecka M, Wulf HC, Nielsen OF, Christensen DH, Hercogova J (1997) Distinctive molecular abnormalities in benign and malignant skin lesions: studies by Raman spectroscopy. Photochem Photobiol 66(4): 418–423 9337612 10.1111/j.1751-1097.1997.tb03167.x

[bib15] Hattori Y, Komachi Y, Asakura T, Shimosegawa T, Kanai G, Tashiro H, Sata H (2007) *In vivo* Raman study of the living Rat esophagus and stomach using a micro-Raman probe under an endoscopy. Appl Spectrosc 61(6): 579–584 17650367 10.1366/000370207781269747

[bib16] Huang Z, Lui H, Chen XK, Alajlan A, McLean DI, Zeng H (2004) Raman spectroscopy of *in vivo* cutaneous melanin. J Biomed Opt 9(6): 1198–1205 15568940 10.1117/1.1805553

[bib17] Huang Z, Lui H, McLean DI, Korbelik M, Zeng H (2005) Raman spectroscopy in combination with near-infrared autofluorescence background enhances the *in vivo* assessment of malignant tissues. Photochem Photobiol 81(5): 1219–1226 15869327 10.1562/2005-02-24-RA-449

[bib18] Huang Z, McWilliams A, Lui H, McLean DI, Lam S, Zeng H (2003) Near-infrared Raman spectroscopy for optical diagnosis of lung cancer. Int J Cancer 107: 1047–1052 14601068 10.1002/ijc.11500

[bib19] Huang Z, Zeng H, Hamzavi I, McLean DI, Lui H (2001) Rapid near-infrared Raman spectroscopy system for real-time *in vivo* skin measurements. Opt Lett 26: 1782–1784 18059697 10.1364/ol.26.001782

[bib20] Kumar KK, Anand A, Chowdary MVP, Thakur K, Kurien J, Krishna CM, Mathew S (2007) Discrimination of normal and malignant stomach mucosal tissues by Raman spectroscopy: a pilot study. Vib Spectrosc 44: 382–387

[bib21] Lachenbruch P, Mickey RM (1968) Estimation of error rates in discriminant analysis. Technometrics 10: 1–11

[bib22] Lau DP, Huang Z, Lui H, Anderson DW, Berean K, Morrison MD, Shen L, Zeng H (2005) Raman spectroscopy for optical diagnosis in the larynx – preliminary findings. Lasers Surg Med 37(3): 192–200 16127671 10.1002/lsm.20226

[bib23] Lauwers GY, Riddell RH (1999) Gastric epithelial dysplasia. Gut 45: 784–790 10517922 10.1136/gut.45.5.784 PMC1727726

[bib24] Ling XF, Xu YZ, Weng SF, Li WH, Xu F, Hammaker RM, Fateley WG, Wang F, Zhou XS, Soloway RD, Ferraro JR, Wu JG (2002) Investigation of normal and malignant tissue samples from the human stomach using Fourier transform Raman spectroscopy. Appl Spectrosc 56(5): 570–573

[bib25] Mahadevan-Jansen A, Mitchell MF, Ramanujam N, Malpica A, Thomsen S, Utzinger U, Richards-Kortum R (1998a) Near-infrared Raman spectroscopy for *in vitro* detection of cervical precancers. Photochem Photobiol 68: 123–132 9679458 10.1562/0031-8655(1998)068<0123:nirsfv>2.3.co;2

[bib26] Mahadevan-Jansen A, Mitchell MF, Ramanujam N, Utzinger U, Richards-Kortum R (1998b) Development of a fiber optic probe to measure NIR Raman spectra of cervical tissue *in vivo*. Photochem Photobiol 68: 427–431 9747597

[bib27] Mahadevan-Jansen A, Richards-Kortum R (1996) Raman spectroscopy for the detection of cancers and precancers. J Biomed Opt 1: 31–70 23014644 10.1117/12.227815

[bib28] Mizuno A, Kitajima H, Kawauchi K, Muraishi S, Ozaki Y (1994) Near-infrared Fourier transform Raman spectroscopic study of human brain tissues and tumors. J Raman Spectrosc 25: 25–29

[bib29] Molckovsky A, Song LM, Shim MG, Marcon NE, Wilson BC (2003) Diagnostic potential of near-infrared Raman spectroscopy in the colon: differentiating adenomatous from hyperplastic polyps. Gastrointest Endosc 57: 396–402 12612529 10.1067/mge.2003.105

[bib30] Mountford CE, Somorjai RL, Malycha P, Gluch L, Lean C, Russell P, Barraclough B, Gillett D, Himmelreich U, Dolenko B, Nikulin AE, Smith IC (2001) Diagnosis and prognosis of breast cancer by magnetic resonance spectroscopy of fine-needle aspirates analysed using a statistical classification strategy. Br J Surg 88: 1234–1240 11531873 10.1046/j.0007-1323.2001.01864.x

[bib31] Mourant JR, Short KW, Carpenter S, Kunapareddy N, Coburn L, Powers TM, Freyer JP (2005) Biochemical differences in tumorigenic and nontumorigenic cells measured by Raman and infrared spectroscopy. J Biomed Opt 10(3): 031106 16229631 10.1117/1.1928050

[bib32] Sabet EA, Okai T, Minamoto T, Mai M, Sawabu N (2003) Visualizing the gastric wall with a 30-MHz ultrasonic miniprobe: *ex vivo* imaging of normal gastric sites and sites of early gastric cancer. Abdom Imaging 28(2): 252–256 12592475 10.1007/s00261-002-0035-1

[bib33] Sasic S (2001) Eigenvalues and principal component loadings or heavily overlapped vibrational spectra. Spectrochim Acta A 57: 323–336 10.1016/s1386-1425(00)00386-3 11206567

[bib34] Shetty G, Kendall C, Shepherd N, Stone N, Barr H (2006) Raman spectroscopy: elucidation of biochemical changes in carcinogenesis of oesophagus. Br J Cancer 94: 1460–1464 16622450 10.1038/sj.bjc.6603102 PMC2361283

[bib35] Shim MG, Song LM, Marcon NE, Wilson BC (2000) *In vivo* near-infrared Raman spectroscopy: demonstration of feasibility during clinical gastrointestinal endoscopy. Photochem Photobiol 72: 146–150 10911740 10.1562/0031-8655(2000)072<0146:IVNIRS>2.0.CO;2

[bib36] Stone N, Kendall C, Sheperd N, Crow P, Barr H (2002) Near-infrared Raman spectroscopy for the classification of epithelial pre-cancers and cancers. J Raman Spectrosc 33: 564–573

[bib37] Stone N, Kendall C, Smith J, Crow P, Barr H (2004) Raman spectroscopy for identification of epithelial cancers. Faraday Discuss 126: 141–157 14992404 10.1039/b304992b

[bib38] Stone N, Stavroulaki P, Kendall C, Birchall M, Barr H (2000) Raman spectroscopy for early detection of laryngeal malignancy: preliminary results. Laryngoscope 110: 1756–1763 11037840 10.1097/00005537-200010000-00037

[bib39] Teh M, Tan KB, Seet BL, Yeoh KG (2002) Study of p53 immunostaining in the gastric epithelium of CagA-positive and CagA-negative helicobacter pylori gastritis. Cancer 95(3): 499–505 12209741 10.1002/cncr.10697

[bib40] Thomas Jr GJ, Prescott B (1977) Secondary structure of histones and DNA in chromatin. Science 197(4301): 385–388 560060 10.1126/science.560060

[bib41] Utzinger U, Heintzelman DL, Mahadevan-Jansen A, Malpica A, Follen M, Richards-Kortum R (2001) Near-infrared Raman spectroscopy for *in vivo* detection of cervical precancers. Appl Spectrosc 55: 955–959

